# Nitric Oxide Alleviated Arsenic Toxicity by Modulation of Antioxidants and Thiol Metabolism in Rice (*Oryza sativa* L.)

**DOI:** 10.3389/fpls.2015.01272

**Published:** 2016-01-12

**Authors:** Amit P. Singh, Garima Dixit, Amit Kumar, Seema Mishra, Pradyumna K. Singh, Sanjay Dwivedi, Prabodh K. Trivedi, Debasis Chakrabarty, Shekhar Mallick, Vivek Pandey, Om P. Dhankher, Rudra D. Tripathi

**Affiliations:** ^1^C.S.I.R.-National Botanical Research InstituteLucknow, India; ^2^Stockbridge School of Agriculture, University of Massachusetts AmherstAmherst, MA, USA

**Keywords:** arsenate, arsenic transporter, iron transporter, nitric oxide, non-protein thiol, rice

## Abstract

Nitric oxide (NO) is a gaseous signaling molecule and has a profound impact on plant growth and development. It is reported to serve as pro oxidant as well as antioxidant in plant system. In the present study, we evaluated the protective role of NO against arsenate (As^V^) toxicity in rice plants. As^V^ exposure has hampered the plant growth, reduced the chlorophyll content, and enhanced the oxidative stress, while the exogenous NO supplementation has reverted these symptoms. NO supplementation has reduced the arsenic (As) accumulation in root as well as shoot. NO supplementation to As^V^ exposed plants has reduced the gene expression level of *OsLsi1* and *OsLsi2*. As^V^ stress significantly impacted thiol metabolism, it reduced GSH content and GSH/GSSG ratio, and enhanced the level of PCs. NO supplementation maintained the GSH/GSSG ratio and reduced the level of PCs. NO supplementation reverted As^V^ induced iron deficiency in shoot and had significant impact of gene expression level of various iron transporters (*OsYSL2, OsFRDL1, OsIRT1*, and *OsIRO2*). Conclusively, exogenous application of NO could be advantageous against As^V^ toxicity and could confer the tolerance to As^V^ stress in rice.

## Introduction

Arsenic (As) is ubiquitous element in the earth crust and present in almost all type of soils. As and its compounds are classified as Group 1 carcinogens by International Agency for Research on Cancer. High doses of As can cause death, but chronic lower level exposures result in serious health problems such as skin lesions and cancer (Kumar et al., 2015). As contamination in drinking water is main source of As exposure for humans and when this As contaminated water is used for irrigation of crops and fodder it becomes part of food chain (Finnegan and Chen, 2012). Bangladesh and West Bengal (India) are extremely high As contaminated regions where As concentration in water has been reported up to 3200 μgL^-1^ against the safe limit of 10 μgL^-1^ recommended by WHO (McCarty et al., 2011). As exists as inorganic as well as organic form in environment. Arsenate (As^V^) and arsenite (As^III^) are two principal forms of inorganic As. As^V^ form predominates in aerobic soil while under reduced condition As^III^ form predominates. Rice is grown under flooded conditions where As^III^ form dominates. Further, most of the As taken up by the plants is also reduced and stored as As^III^. The two inorganic forms of As; As^III^ and As^V^ gets entry in to plant system through aquaporins and phosphate transporters, respectively (Tripathi et al., 2007; Dixit et al., 2015a). Two well known aquaporin transporters of As^III^ in rice are *OsLsi1*, that is responsible for uptake of As^III^ in to root cells from external medium and *OsLsi2* is a eﬄux transporter and responsible for As^III^ accumulation in shoot and grain (Ma et al., 2008).

Arsenic is non-essential toxic element for plant growth and development. Rice is an efficient accumulator of As and unfortunately, major output of rice comes from these As contaminated regions thus elevated As accumulation in rice may become disaster (Kumar et al., 2015). As also affects the amino acid profile and elemental content of grain that reduces the nutritional value of rice (Kumar et al., 2014a). Numerous physiological processes in plant system are susceptible for As toxicity (Srivastava et al., 2015). As exposure induces reactive oxygen species (ROS) synthesis which leads to cellular membrane damage (Kumar et al., 2013, 2014b). To cope with enhanced level of oxidative stress plants are equipped with antioxidant system that gets activated under As stress conditions (Gupta and Ahmad, 2014; Singh et al., 2015). As induces the synthesis of phytochelatins (PCs) that bind to As^III^ and sequester it into vacuole and reduces the free As in cytoplasm (Dixit et al., 2015b).

Nitric oxide (NO) is a gaseous free radical molecule and serves as an effective signaling molecule in plant system. NO plays a crucial role in immune response against pathogen attack in plants (Bellin et al., 2013). Exogenously supplied NO has been demonstrated to provide a protection against heavy metals such as copper (Yu et al., 2005), aluminum (Sun et al., 2014), manganese (Srivastava and Dubey, 2012), As (Singh et al., 2009), and cadmium (Singh et al., 2008). NO can neutralize heavy metal induce ROS in two ways, first being a free radical it can directly react with ROS and neutralize them (Laspina et al., 2005) and second being a signaling molecule, it may stimulate antioxidant system to abate oxidative stress (Lamattina et al., 2003; Laspina et al., 2005). NO brings the post translational modification of proteins by nitrosylating their cysteine residue. Ascorbate peroxidase (APX), catalase (CAT), and superoxide dismutase (SOD) are good candidates for NO regulated antioxidants in plants (Groß et al., 2013). NO is associated with iron (Fe) homeostatic and mediates the Fe dependent ferritin expression in *Arabidopsis* (Murgia et al., 2002). Under Fe deficient conditions NO is rapidly produced in roots and activates Fe starvation pathways (Graziano and Lamattina, 2007). Exogenous NO also have profound impact on genes involved in Fe uptake (Koen et al., 2012).

Plants follow two different mechanisms for iron (Fe) acquisition; Strategy I in non-graminaceous plants and Strategy II in graminaceous plants (Römheld and Marschner, 1986). In Strategy I, ferric chelates (Fe^+3^ chelate) are reduced in to ferrous (Fe^+2^) ions at the root surface and so generated Fe^+2^ ions are absorbed across the plasma membrane (Kobayashi and Nishizawa, 2012). Rice follows Strategy II for Fe uptake, where plant roots secrete mugineic acid (MA) that form Fe^+3^-MA complex which is taken up by root cells by YSL transporters (Kobayashi and Nishizawa, 2012). Various transporters such as OsFRDL1, OsYSL2, and OsNRAMP5 are involved for Fe transport in rice through Strategy II. Fe^+2^ is abundant form of Fe in submerged and anaerobic conditions thus to uptake Fe^+2^ rice plant has a unique transporter OsIRT1, which facilitates this crop to absorb Fe^+2^ directly, however, it is the unique feature of Strategy I plants (Kobayashi and Nishizawa, 2012). OsIRO2 is key regulator of various Fe transporters (Ogo et al., 2007). OsFRDL1 is expressed in rice root pericycle and encodes citrate eﬄuxer, that is required for efficient Fe translocation (Yokosho et al., 2009) and OsYSL2 is responsible for long distance transport of chelated Fe^+3^ to sink tissues (Ishimaru et al., 2010).

The present study aims to investigate the role of exogenously supplied NO (SNP as NO donor) for alleviation of As^V^ toxicity in hydroponically grown rice. SNP releases NO in the form of nitrosonium cation (NO^+^) on its reaction with thiolic legends (RSH). An electron-transfer process is key step, which leads to the formation of the reduced SNP radical and the corresponding *S*-nitrosothiol, that is storage form of NO (Grossi and D’Angelo, 2005). NO release from SNP also largely depends upon light intensity (Lum et al., 2005). The study elucidates impact of exogenously supplied NO on antioxidants, non-protein thiol (NPT) metabolism, accumulation of As and Fe and expression of various Fe and As^III^ transporters against moderate (25 μM) and high (50 μM) doses of As^V^.

## Methods and Materials

### Growth Conditions and Experimental Design

Seeds of *Oryza sativa* cv. Jaya collected from Masina Research Centre, Pvt. Ltd., Bihar (India), were surface sterilized using 10% H_2_O_2_ for 30 s and washed with Milli-Q water. Seeds were germinated on moist pre-sterilized blotting sheets layered on a tray in seed germinator for 4 days at 25°C and relative humidity was 65%. After 7 days, uniform size seedlings were selected and placed in 150 ml beakers, covered with black sheet, containing 100 ml of 100% Hewitt nutrient medium, prepared in Milli-Q water (pH 6.8–7.0) and grown for another 10 days under light intensity 210 μM cm^-2^ s^-1^ (16/8 h; day/night) before treatment. After 10 days of growth in nutrient medium, treatments were provided as As^V^ (25 and 50 μM) using the salt Na_2_HAsO_4_ and NO (100 μM) using salt sodium nitroprusside (SNP, a NO donor) for 7 days. Plants treated by 25 or 50 μM As^V^ or 100 μM SNP are abbreviated as As^V^25, As^V^50, and NO, respectively. Plants treated with As^V^25 or As^V^50 supplemented with NO are abbreviated as NO + As^V^25 and NO + As^V^50. Plants grown only in Hewitt solution served as control. In the present study, SNP is used as efficient NO donor because it give rise to a persistent pattern of NO generation than other NO donors (Mur et al., 2013).

### Estimation of Photosynthetic Pigments

For chlorophyll estimation, 100 mg fresh leaves were crushed in 5 ml of 80% chilled acetone and homogenate was centrifuged at 10,000 × *g* for 10 min. Chlorophyll and carotenoid content in supernatant was estimated as described by the method of Arnon (1949) and Duxbury and Yentsch (1956), respectively.

### Estimation of Lipid Peroxidation (MDA) and Hydrogen Peroxide

For MDA and H_2_O_2_ estimation, 300 mg fresh leaves or roots were crushed in 3 ml of 0.2% trichloroacetic acid and homogenate was centrifuged at 10,000 × *g* for 10 min. and supernatant was collected for further estimation. MDA and H_2_O_2_ contents were estimated as described by Heath and Packer (1968) and Velikova et al. (2000), respectively.

### Determination of Antioxidant Enzymes and Nitrate Reductase Activities and Nitrite Level

For analysis of enzyme activities, 300 mg of fresh leaves or roots were ground in liquid N_2_, and extracted with 3 ml of ice cold 100 mM potassium phosphate buffer (pH 7.8) having 1% (w/v) polyvinylpyrrolidone (PVP). The homogenate was centrifuged at 8000 × *g* at 4°C for 15 min and supernatant was used for enzyme assays. The activity of SOD was measured by the method of Beauchamp and Fridovich (1971), APX by the method of Nakano and Asada (1981), GPX by the method of Kato and Shimizu (1987), and CAT by the method of Scandalios et al. (1983). The activity of nitrate reductase (NR) and the level of nitrite were determined by the method of Hageman and Reed (1980).

### Estimation of Non-protein Thiol Metabolites and Related Enzymes

The level of GSH and GSSG was measured by following the method of Hissin and Hilf (1976). NPT content was measured by following the method of Ellman (1959). The concentration of PCs was calculated as PCs = NPT - (GSH + GSSG; Duan et al., 2011).

Assay of cysteine synthase (CS) and γ-glutamylcysteine synthetase (γ-ECS) activities, was performed following the method of Seelig and Meister (1985) and Saito et al. (1994), respectively. The GR activity was assayed by following Smith et al. (1988). Glutathione-*S*-transferase (GST; EC 2.5.1.18) activity was assayed following Habig and Jakoby (1981). Estimation of Cysteine was performed using acid ninhydrin reagent by the method of Gaitonde (1967).

### Nitric Oxide and Reactive Oxygen Species Imaging

For NO detection roots were incubated for 1 h at 25°C, in darkness, with 10 mM DAF-2DA (Calbiochem; excitation at 495 nm, emission at 515 nm) prepared in 10 mM Tris-HCl (pH 7.4), as described by Sandalio et al. (2008).

For ROS detection roots were incubated with 25 mM H_2_DCF-DA (Calbiochem; excitation at 485 nm, emission at 530 nm) for 1 h in darkness at 25°C as described by Rodríguez-Serrano et al. (2006). Then roots were washed three times for 10 min each with same buffer and fluorescence was visualized by confocal microscope, Zeiss LSM510 Meta.

### Elemental Analysis

Element analysis was carried out by method of Mallick et al. (2013). Briefly, plant tissues root (300 mg) and shoot (500 mg) were oven dried at 70°C and digested in HNO_3_:HCl (3:1). Digested samples were filtered through Whatman filter paper 42 and volume was made to 10 ml by Milli-Q water. As and Fe were estimated by using AAS (GBC Avanta S, USA) fitted with a hydride generator (MDS2000) using NaH_2_BO_4_ + NaOH (3 M) and HCl (3 M). The values were presented in μg per gram dry weight (μg g^-1^dw).

### Gene Expression Analysis Using Quantitative RT-PCR

Approximately 5 μg, RNase free DNase-treated, total RNA isolated from roots of rice plants was reverse-transcribed using SuperScriptII (Fermentas, USA), following the manufacturer’s recommendation. The synthesized cDNA was diluted 1:5 in DEPC water and subjected to quantitative RT-PCR (qRT-PCR) analysis. The qRT-PCR was performed using an ABI 7500 instrument (ABI Biosystems, USA) using primers listed in Supplementary Table S1. Each qPCR reaction mixture contained 5 μl of SYBR Green Supermix (ABI Biosystems, USA), 1 μl of the diluted cDNA reaction mixture (corresponding to 5 ng of starting amount of RNA) and 10 pM of each primer in a total reaction volume of 10 μl. The qPCR reactions were performed under following conditions: 10 min at 95°C and 40 cycles of the one step thermal cycling of 3 s at 95°C and 30 s at 60°C in a 96-well reaction plate. Actin gene was used as an internal control to estimate the relative transcript levels of the target gene. Specificity of amplicons generated in qPCR reactions was verified by melt curve analysis. Each qPCR reaction was performed in triplicate (technical replicates) for each biological replicate (three for each treatment). Relative gene expression was calculated using ^ΔΔ^CT method of Livak and Schmittgen (2001).

### Statistical Analysis and Analytical Quality Control

The whole experiment was set up in the randomized block design. The data were subjected to Duncan’s Multiple Range Test (DMRT) for the analysis of significant difference between the treatments. Analytical data quality of the elements was ensured through repeated analysis (*n* = 6) of Standard Reference Material. Standard Certified reference material (CRM 028-050), procured from Resource Technology Corporation, USA (Lot no. IH 028), was used to check accuracy of the AAS. The blanks were run all the time to eliminate the background noise.

## Results

### Morphology

Arsenate exposure hampered the plant growth and showed various symptoms of toxicity, such as chlorosis, growth inhibition, and necrosis (data not shown). In As^V^ exposed plants, root hairs growth was hampered while, NO supplementation to As^V^ exposed plants, reverted the root hairs growth and it was comparable to that of control plants (Supplementary Figure S1). A dose dependent significant decrease was observed in root, shoot length and biomass in As^V^ exposed plants. As^V^ exposed plants supplemented with NO showed better growth than As^V^ alone stressed plants. Root, shoot length, and biomass were comparable with that of control in As^V^ + NO treatments. As^V^ stress has reduced the total chlorophyll content 22 and 35% in As^V^25 and As^V^50 treated plants, respectively. NO supplementation to As^V^ stressed plants overcame the As^V^ induced chlorophyll decay and total chlorophyll content was comparable to that of control. As^V^ and NO both had no significant impact on carotenoid content (**Table 1**).

**Table 1:** Effect on shoot, root lengths (cm), fresh-weight (g), total chlorophyll content (mg g^-1^fw), and carotenoid content (mg g^-1^ fw) of rice after 7 days of treatment with different combinations of NO and As^V^.

Treatments	Root length	Shoot length	Biomass	Total Chlorophyll	Carotenoids
Control	4.18^cd^ ± 0.63	30.56^d^ ± 0.58	0.33^ab^ ± 0.10	2.18^c^ ± 0.04	0.153^a^ ± 0.004
NO	4.50^d^ ± 0.39	35.52^e^ ± 1.21	0.47^b^ ± 0.09	2.41^d^ ± 0.08	0.156^a^ ± 0.004
As^V^25	3.13^b^ ± 0.63	24.78^b^ ± 0.50	0.21^a^ ± 0.08	1.69^b^ ± 0.06	0.160^a^ ± 0.005
As^V^50	2.25^a^ ± 0.29	22.25^a^ ± 0.50	0.15^a^ ± 0.07	1.40^a^ ± 0.11	0.160^a^ ± 0.004
NO + As^V^25	4.00^bcd^ ± 0.45	31.65^d^ ± 0.90	0.26^ab^ ± 0.12	2.32^d^ ± 0.01	0.159^a^ ± 0.001
NO + As^V^50	3.50^bc^ ± 0.41	28.50^c^ ± 1.29	0.24^a^ ± 0.17	2.14^c^ ± 0.04	0.156^a^ ± 0.003

*Values marked with same alphabets are not significantly different (DMRT, *p* < 0.05). All the values are means of five replicates ±SD.*

### Endogenous Nitric Oxide

**FIGURE 1:**
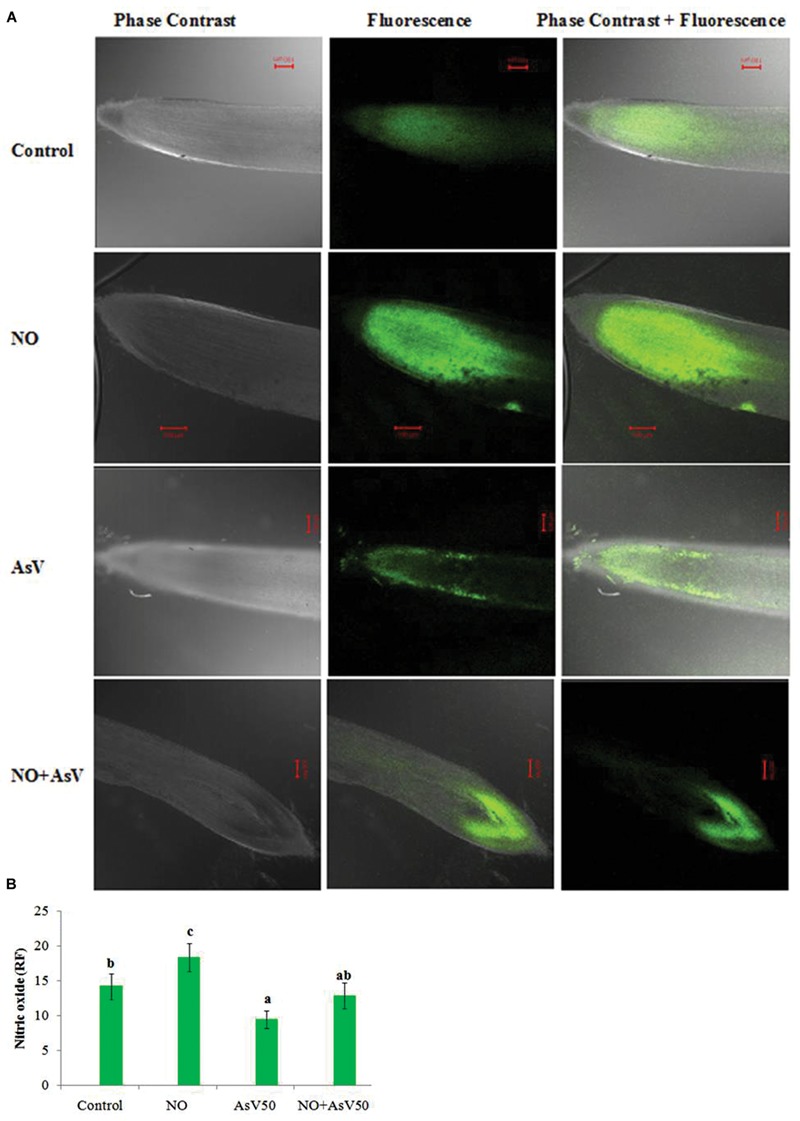
**Imaging of NO production in *Oryza sativa* by CLSM.** Images are showing the NO-dependent DAF-FM 2DA **(A)** fluorescence (green; excitation at 495 nm, emission at 515 nm) after 7 days treatment with different combinations of NO and As^V^ and **(B)** relative fluorescence.

Endogenous level of NO in root was estimated by NO mediated fluorescence. Exogenous application of NO enhanced the fluorescence than control root. In As^V^ exposed plants NO dependent fluorescence was reduced than control while, NO supplementation to As^V^ stressed plants has enhanced the fluorescence than alone As^V^ stressed root (**Figures 1A,B**).

### Oxidative Stress

Endogenous accumulation of ROS in root was carried out by H_2_DCF-DA staining. NO treatment did not altered ROS dependent fluorescence significantly while, As^V^ exposure has enhanced drastically. NO supplementation to As^V^ exposed plants reduced the ROS dependent fluorescence than As^V^ exposed roots (Supplementary Figures S2A,B).

**FIGURE 2:**
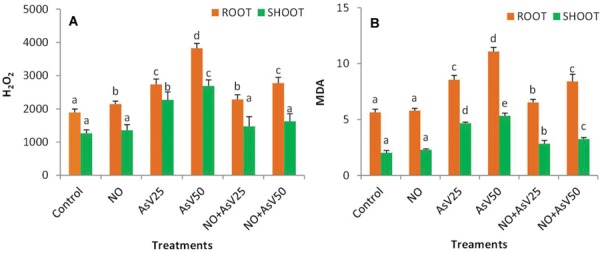
**Effect on **(A)** H_2_O_2_ (nMol g^-1^fw) and **(B)** MDA (mMol g^-1^fw) in the rice after 7 days of treatment with different combinations of As^V^ and NO.** Values marked with same alphabets are not significantly different (DMRT, *p* < 0.05). All the values are mean of three replicates ±SD.

Arsenate stress enhanced H_2_O_2_ content by 1.8- and 2.1-fold in shoot and 1.4- and 2.0-fold in root at As^V^25 and at As^V^50, respectively, than control. NO supplementation to As^V^ stressed plants reduced the H_2_O_2_ accumulation by 35 and 16% in NO + As^V^25 and 39 and 27% in NO + As^V^50 in shoot and root, respectively, than corresponding As^V^ alone exposed plants. As^V^ stress also caused enhanced lipid peroxidation, measured in terms of MDA. The level of MDA enhanced to 2.3- and 2.6-fold in shoot and 1.5- and 2.0-fold in root with increase in As^V^ exposure concentration (As^V^25 and As^V^50) in comparison to control. NO supplementation to As^V^ stressed plants reduced the level of MDA significantly in both root and shoot than corresponding As^V^ alone exposed plants (**Figures 2A,B**).

### Antioxidant Enzymes

**FIGURE 3:**
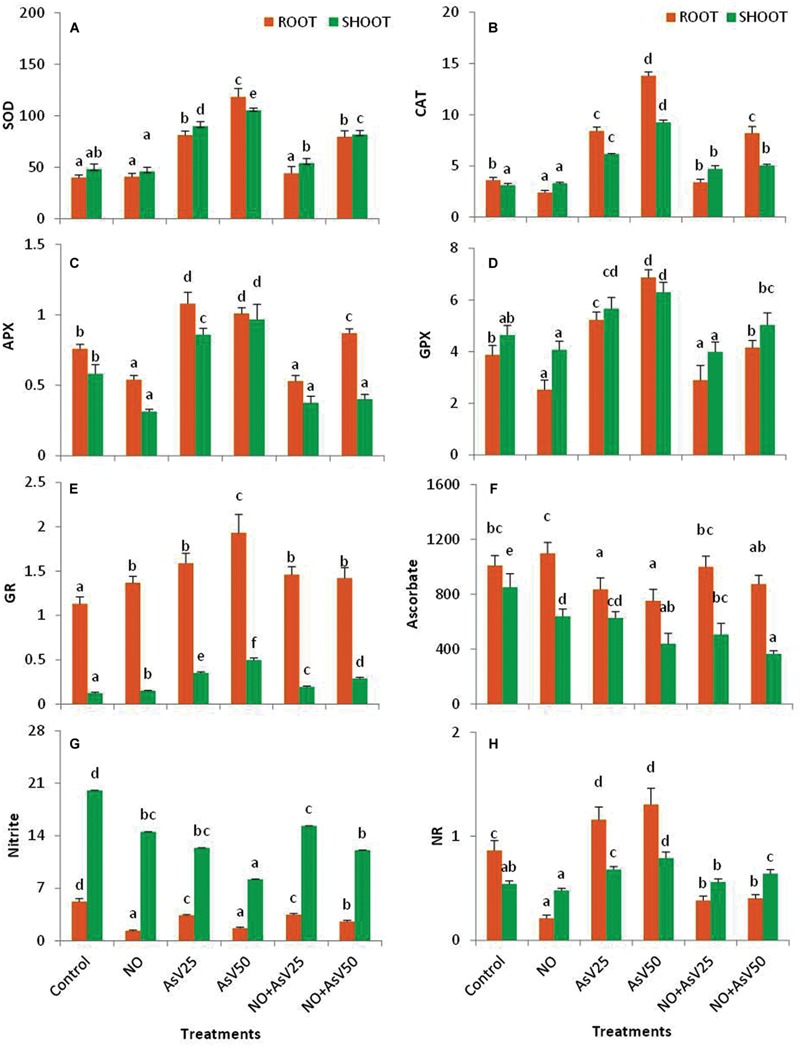
**Effect on **(A)** SOD (U g^-1^fw), (B) CAT (mMol min^-1^ g^-1^fw), **(C)** APX (mMol min^-1^ g^-1^fw), **(D)** GPX (mMol min^-1^ g^-1^fw), **(E)** GR (U mg^-1^P), **(F)** Ascorbic acid (μMol g^-1^fw), **(G)** Nitrite (μmol g^-1^fw) and **(H)** NR (μMol KNO_2_ formed min^-1^ g^-1^fw) in the rice after 7 days of treatment with different combinations of NO and As^V^.** Values marked with same alphabets are not significantly different (DMRT, *p* < 0.05). All the values are means of four replicates ±SD.

Arsenate stress has enhanced the SOD activity by ca. 1.9- and 2.2-fold in shoot and ca. twofold and threefold in root in dose dependent manner than control. Supplementation of NO alone has no significant impact on SOD activity. NO supplementation to As^V^ stressed plants significantly reduced the SOD activity than corresponding As^V^ alone treated plants (**Figure 3A**).

Nitric oxide treatment reduced CAT and GPX activity by 33 and 34%, respectively, in root while no significant impact was observed in shoot in comparison to control. APX activity was also reduced by 29 and 46% in root and shoot, respectively, in NO treated plants than control. In As^V^ stressed plants, CAT activity was enhanced ca. twofold and threefold in shoot and ca. 2.3- and 3.9-fold in root, and GPX activity was enhanced to 22 and 36% in shoot and 35 and 77% in root, at As^V^25 and As^V^50, respectively, than control. APX activity was also increased upon As^V^ exposure both in root and shoot than control. NO supplementation to As^V^ stressed plants resulted in significant reduction in the activities of CAT, APX, and GPX both in root and shoot than corresponding As^V^ alone exposed plants (**Figures 3B–D**). In contrast, the activity of GR was enhanced both by NO and As^V^ alone treated plants both in root and shoot. However, NO supplementation to As^V^ stressed plants, reduced the GR activity than As^V^ alone stressed plants (**Figure 3E**).

Nitric oxide treatment has reduced the ascorbate content in shoot (25%), while no significant change was observed in root than control. As^V^ exposure also reduced the ascorbate content both in root and shoot in dose dependent manner. NO supplementation to As^V^ stressed plants further reduced the ascorbate content in shoot, while in root it enhanced to control levels (**Figure 3F**).

Nitric oxide and As^V^ alone has reduced the nitrite content in both root and shoot than control with more decline in root. NO treatment also reduced the NR activity to one fourth in root, while As^V^ stress enhanced the NR activity both in root and shoot in dose dependent manner than control. NO supplementation to As^V^ stressed plants enhanced the nitrite content in shoot, while NR activity was reduced both in root and shoot than corresponding As^V^ alone stressed plants both in root and shoot (**Figures 3G,H**).

### Thiol Metabolism

**FIGURE 4:**
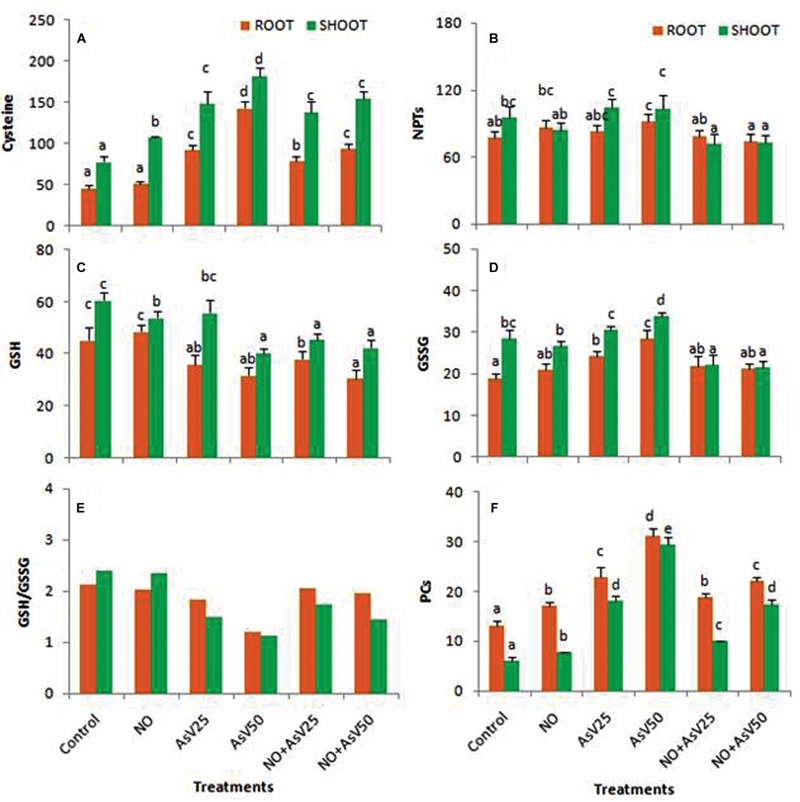
**Effect on **(A)** Cysteine (nMol g^-1^fw), **(B)** NPT (μMol g^-1^fw), (C) GSH (μMol g^-1^fw), **(D)** GSSG (μMol g^-1^fw), **(E)** Ratio of GSH/GSSG, **(F)** Phytochelatins (PCs; μMol g^-1^fw)in the rice after 7 days of treatment with different combinations of NO and As^V^.** Values marked with same alphabets are not significantly different (DMRT, *p* < 0.05). All the values are means of four replicates ±SD.

Arsenate and NO alone treatment enhanced the cysteine content in both root and shoot in dose dependent manner than control. NO supplementation to As^V^ stressed plants has reduced the cysteine content at higher As^V^ concentration, i.e., at 50 μM, than corresponding As^V^ alone stressed plants though it was still higher than control levels (**Figure 4A**). NO and As^V^ treatment did not caused much changes in NPT level both in root and shoot than control. NO supplementation to As^V^ stressed plants slightly reduced the NPT content than As^V^ alone stressed plants (**Figure 4B**). Plants exposed to higher concentration of As^V^ (As^V^50) has decrease the GSH content in both root and shoot significantly than control. Exposure to higher concentration of As^V^ (As^V^50) has enhanced the GSSG level in both root and shoot, while the lower concentration (As^V^25) enhanced the GSSG level only in root than control. NO supplementation to As^V^ stressed plants has reduced the GSSG level both in root and shoot than alone As^V^ exposed plants. NO treatment has no significant impact on GSH/GSSG ratio although As^V^ has reduced the ratio significantly in dose dependent manner than control in both root and shoot, although, more reduction was observed in root than shoot. NO supplementation to As^V^ stressed plants significantly enhanced the GSH/GSSG ratio than corresponding alone As^V^ stressed plants (**Figures 4C–E**). NO treatment enhanced the PCs level by approximately 25% in both root and shoot than control plants. As^V^ exposure also enhanced the PCs level significantly both in root and shoot in dose dependent manner. NO supplementation to As^V^ significantly reduced the PCs level than alone As^V^ stressed plants (**Figure 4F**).

**FIGURE 5:**
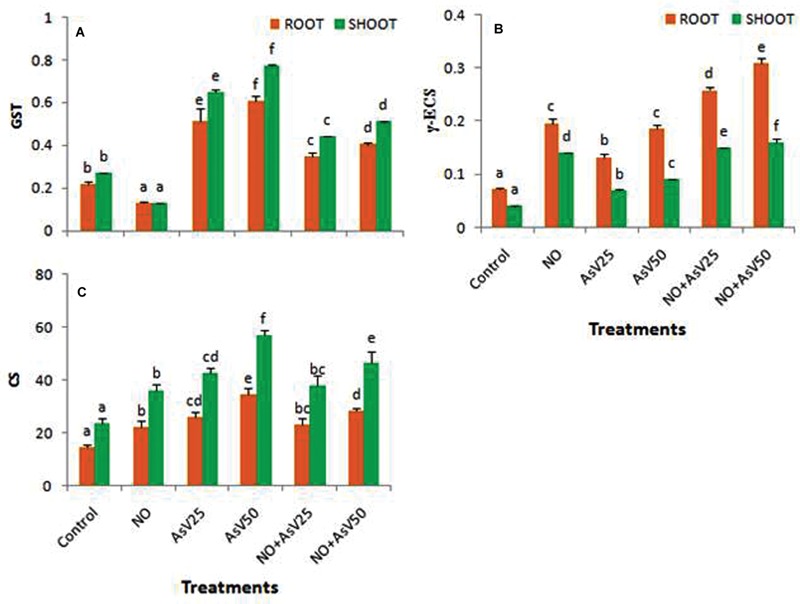
**Effect on **(A)** GST (U mg^-1^ P), **(B)** γ ECS (U mg^-1^ P) and **(C)** Cysteine synthase [nMol (cys) min^-1^ g^-1^fw] in the rice after 7 days of treatment with different combinations of NO and As^V^.** Values marked with same alphabets are not significantly different (DMRT, *p* < 0.05). All the values are means of four replicates ±SD.

Glutathione-*S*-transferase activity was reduced to approximately half in NO treated plants than control in both root and shoot. In As^V^ exposed plants, GST activity was enhanced in dose dependent manner than in control in both the root and shoot. NO supplementation to As^V^ stressed plants reduced the GST activity than alone As^V^ stressed plants (**Figure 5A**). γ-ECS activity was enhanced by As^V^ and NO both whether alone or in combination. NO treatment had more significant impact on γ-ECS activity (**Figure 5B**). Similar trend was observed for CS activity (**Figure 5C**).

### Element Accumulation

Arsenate treatment has enhanced the Fe accumulation in root, but reduced in shoot in dose dependent manner. NO treatment has enhanced the Fe accumulation in root (17%) and in shoot (25%) than control. NO supplementation to As^V^ stressed plants, enhanced the Fe accumulation in shoot than corresponding alone As stressed plants. As^III^ exposed plants accumulated a significant amount of As in root and shoot. NO supplementation to As^V^ stressed plants has reduced the As accumulation in root more 30% and in shoot more than 47% than corresponding alone As^V^ exposed plants (**Table 2**).

**Table 2:** Accumulation (μg g^-1^dw) of As and Fe in the roots and shoots of rice after 7 days of treatment with different combinations of NO and As^V^.

Treatments	As Root	As Shoot	Fe Root	Fe Shoot
Control	-	–	265.1^a^ ± 26.4	71.4^cd^ ± 5.6
NO	-	–	311.2^b^ ± 22.8	89.1^e^ ± 6.9
As^V^25	778.1^b^ ± 21.2	86.8^c^ ± 3.8	494.5^d^ ± 26.3	57.8^ab^ ± 4.7
As^V^50	898.2^c^ ± 38.9	92.9^c^ ± 8.6	578.3^e^ ± 31.3	51.3^a^ ± 4.8
NO + As^V^25	542.6^a^ ± 17.2	39.0^a^ ± 2.1	383.9^c^ ± 18.6	79.9^d^ ± 2.2
NO + As^V^50	582.5^ab^ ± 12.4	49.1^b^ ± 2.7	413.4^c^ ± 14.0	66.3^bc^ ± 3.6

*Values marked with same alphabets are not significantly different (DMRT, *p* < 0.05). All the values are means of four replicates ±SD.*

### Arsenite and Fe Transporters

**FIGURE 6:**
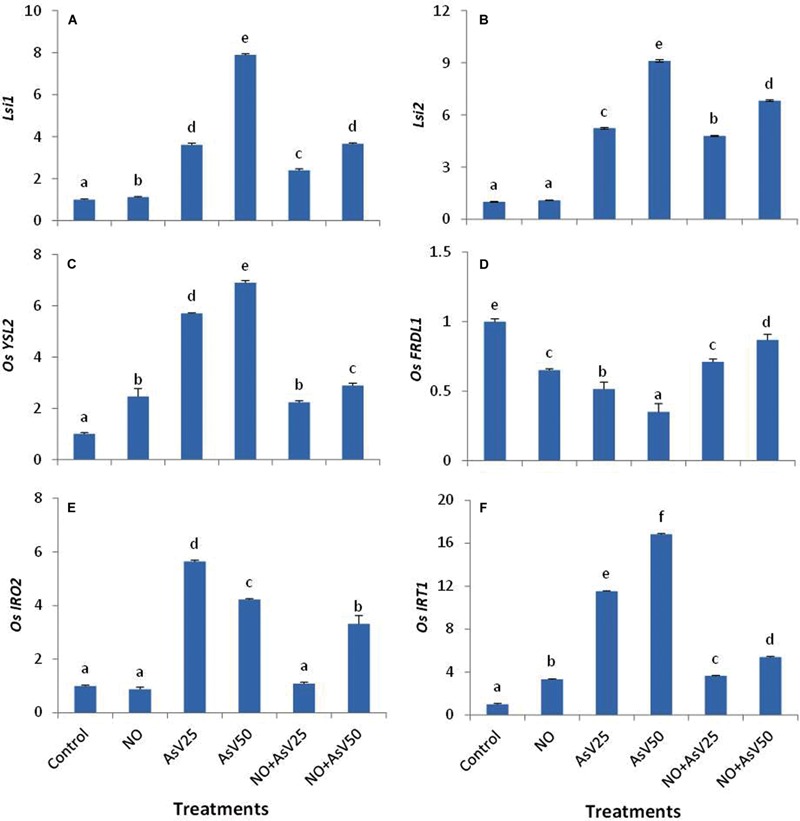
**Relative expression of **(A)***OsLsi1*, **(B)***OsLsi2*, **(C)***OsYSL2*, **(D)***OsFRDL1*, **(E)***OsIRO2*, and **(F)***OsIRT1* in the rice root after 7 days of treatment with different combinations of NO and As^V^.** Values marked with same alphabets are not significantly different (DMRT, *p* < 0.05). All the values are means of four replicates ±SD.

Nitric oxide alone treatment has no significant impact on *OsLsi1* and *OsLsi2* expression level than control. As^V^ exposure has enhanced the expression level of *OsLsi1* and *OsLsi2* in dose dependent manner than control. NO supplementation to As^V^ stressed plants reduced the expression level of these transporters than corresponding alone As^V^ exposed plants (**Figures 6A,B**).

The expression of *OsYSL2* was enhanced upon NO treatment. As^V^ also enhanced the expression of *OsYSL2* in dose dependant manner with up to sevenfold higher expression at As^V^50. NO supplementation to As^V^ stressed plants significantly reduced the expression level of *OsYSL2* than corresponding alone As^V^ exposed plants and expression level was comparable to NO alone treated plants. Similar expression pattern was observed for *OsIRT1*. In contrast, the expression level of *OsFRDL1* was reduced both by NO and As^V^ than control while NO supplementation to As^V^ exposed plants enhanced the expression level than alone As^V^ exposed plants. The expression of *OsIRO2* was reduced by NO while it enhanced by As^V^ exposure. When NO and As^V^ are provided in combination (NO + As), expression level of *OsIRO2* was decreased than corresponding alone As^V^ stressed plants (**Figures 6C–F**)

## Discussion

Nitric oxide is an important gaseous signaling molecule in plant system and has been reported to play a crucial role against various heavy metals (as detailed in Introduction). Present experiment is designed to investigate the protective role of NO during As stress.

Arsenic is well known to adversely affect the plant growth and development after its entry to plant system (Gupta et al., 2009; Dixit et al., 2015c; Pandey and Gupta, 2015). In the hydroponic medium under anaerobic condition most of As^V^ gets converted to As^III^. Xu et al. (2007) observed that 97% of As^V^ supplied in nutrient medium (hydroponic conditions) was converted to As^III^ within 8 h. So, in the present work, although As^V^ was supplied in nutrient medium but transporters of As^III^ were analyzed. In the present study, a significant amount of As was accumulated in rice plant on exposure to As and hampered the plant growth. NO supplementation to As^V^ stressed plants significantly reduced the As accumulation in both root and shoot. NO supplementation also reduced the As translocation from root to shoot. As induced enhanced expression of *OsLsi1* and *OsLsi2* was also reduced by NO. The transporter *OsLsi1* and *OsLsi2* are responsible for As^III^ internalization and its root to shoot translocation, respectively. Since As^III^ is the dominant form inside the plant (Tripathi et al., 2007; Singh et al., 2015) and also probably the main As species translocated to the shoots. Therefore, down regulation of *OsLsi1* and *OsLsi2* would negatively affect the As accumulation. From correlation analysis between expression of *OsLsi1* and As accumulation in root (*R* = 0.85), it is evident that down regulation of *OsLsi1* must be responsible for reduced As accumulation in root. But the correlation value between As accumulation in shoot and expression level of *OsLsi2* is relatively less significant (*R* = 0.53). NO is also reported to activate the ABC transporters (Grün et al., 2006). The ABC transporters are responsible for vacuolar sequestration of As(III)-PC complexes (Song et al., 2010). So it might be possible that in NO treated plants, most of As accumulated in rice root was sequestered in root vacuole in the form of As(III)-PC complex and less As is transported to shoot. Further in the present study, NO treatment also enhanced the PCs synthesis. Since less accumulation of As in shoot might also affect its accumulation in grain which would have great implications with respect to As toxicity in food chain. NO mediated reduced accumulation of As in rice root and coleoptiles was previously reported by Singh et al. (2009).

In the present study, NO enhanced the plant growth in terms of root, shoot length, and biomass. NO serves as positive growth regulator in plant. Recent research has established that NO is a phytohormone that influences diverse physiological processes in plants (Takahashi and Morikawa, 2014). Low concentration of exogenously supplied NO enhances the plant growth, whereas, no promotive effect was observed at higher concentrations (Leshem, 1996). NO supplementation to As^V^ stressed plants partially restored the plant growth. Previously, Tian and Lei (2006) showed that low concentration of NO promotes the growth of wheat, while high concentration has no significant impact. In the present study, NO supplementation to As^V^ stressed plants overcame As^V^ mediated root hairs growth inhibition. It has been previously reported that NO induces the adventitious root development by means of auxin in cucumber (Pagnussat et al., 2002). NO mediated protective effect previously reported in sunflower against Cd stress (Laspina et al., 2005) and in rice against As stress (Singh et al., 2009). In the present study, a significant loss of chlorophyll was observed in As^V^ stressed plants as previously reported in rice (Singh et al., 2015). In the present study, NO supplementation to As^V^ stressed plants reverted As mediated chlorophyll loss. Exogenous NO treatment has been reported to retard the chlorophyll degradation (Eum et al., 2009). This might be the reason for enhanced chlorophyll upon NO supplementation in As^V^ treated plants. NO mediated enhancement of chlorophyll has been reported in maize (Graziano et al., 2002) and in lettuce (Beligni and Lamattina, 2000). Carotenoids content was found relatively unaltered by exogenous application of NO or As^V^. In the present study As^V^ also reduced the Fe content in shoot that may also be responsible for As^V^ mediated chlorosis, while NO has enhanced the iron content in shoot and reverted the chlorosis. It has been previously reported by Graziano and Lamattina (2007) in tomato that exogenous NO improved the plant growth under Fe deficient conditions by modulation of expression of Fe uptake related genes and by regulation of physiological and morphological adaptive responses. Rice plant belongs to family Poaceae and follows the Strategy II for Fe uptake (Kobayashi and Nishizawa, 2012). *OsYSL2* is responsible for long distance transport of Fe(II)-NA (NA, nicotianamine) and Mn(II)-NA complex to sink tissue (Koike et al., 2004). In the present study, NO treated plants enhanced *OsYSL2* expression level while in As^V^ treated plants it was down regulated. Corresponding changes were observed in Fe accumulation in shoot. OsFRDL1 is expressed in root pericycle and also responsible for long distance transport of Fe (Yokosho et al., 2009). OsFRDL1 is citrate eﬄux transporter. This citrate serves as Fe chelator and forms Fe-chelate, which is transported to plant root by different Fe transporters. So, OsFRDL1 is not directly involve in Fe transport but facilitates Fe transport (Kobayashi and Nishizawa, 2012). *OsFRDL1* was down regulated both in NO and As^V^ treated plants. OsFRDL1 mutants show only mild symptoms of Fe deficiency that suggest that there are alternate chelators for xylem Fe transport (Kobayashi and Nishizawa, 2012). In the present study, there was no correlation between Fe accumulation and transcript level of *OsFRDL1*. OsIRO2 is positive transcription regulator and regulates various other genes related to Fe uptake (Kobayashi and Nishizawa, 2012) and induced by Fe deficiency. In the present study, As^V^ treatment induced Fe deficiency in shoot while the transcript levels of *OsIRO2* increased, probably to increase Fe acquisition. Rice despite being a Strategy II plant, have OsIRT1 that allows this plant to absorb Fe^+2^ that is predominant form of iron under anaerobic and submerged conditions. In NO treated plants, *OsIRT1* level was enhanced also previously reported by Koen et al. (2012) in *Arabidopsis*. In As^V^ treated plants Fe accumulation in root and transcript level of *OsIRT1* were enhanced.

In the present study, As^V^ exposure enhanced oxidative stress that was evident by increased level of ROS, MDA, and H_2_O_2_, however, NO supplementation to As^V^ stressed plants showed a protection against oxidative stress and decreased the level of ROS, H_2_O_2_, and MDA. Similar results were reported by Singh et al. (2009) in rice. NO is stable radical but it can react with other radical such as ROS and can neutralize them (Hill et al., 2010). In the present study, NO might have neutralized ROS and H_2_O_2_, therefore, less membrane damage and reduced MDA. Decreased level of As in root and shoot in NO supplemented As^V^ stressed plants may also responsible for reduced oxidative stress beyond the NO mediated antioxidant action.

In the present study, As^V^ exposure caused a significant reduction in NO dependent fluorescence or endogenous level of NO. A similar decrease in NO dependent fluorescence under Cd stress was also observed in shoot (Rodríguez-Serrano et al., 2009), however, Besson-Bard et al. (2009) reported enhancement in NO dependent fluorescence in both root and shoot in *Arabidopsis thaliana* under Cd stress. During heavy metal exposure endogenous level of NO may increase or decrease depends upon plant species and experimental setup (Arasimowicz-Jelonek et al., 2011). Reduced level of NO in root was also found to correlate with enhanced level of ROS. It again justifies the antioxidant behavior of NO.

Superoxide dismutase activity was enhanced under As^V^ stress as previously reported by Kumar et al. (2013). NO supplementation to As^V^ stressed plants also reduced the SOD activity. Similar results were previously reported by Singh et al. (2009). APX, GPX, and CAT play a crucial role in H_2_O_2_ degradation. Under As^V^ stress, activity of these enzymes was enhanced due to enhanced level of H_2_O_2_. There are contrasting reports on influence of NO on CAT activity. In tobacco plants, NO was reported to inhibit the CAT activity (Clark et al., 2000), while in wheat NO treatment has enhanced the CAT activity (Sun et al., 2014). In the present study, NO treatment has enhanced the CAT activity. This contrasting behavior in CAT activity may be due to use of different NO donors, (Clark et al., 2000) used SNAP (*S*-Nitroso-*N*-Acetyl-*D,L*-Penicillamine) and GSNO (*S*-nitrosoglutathione) as NO donor while (Sun et al., 2014) and in the present study SNP used as NO donor.

Cysteine is precursor of GSH and γ-ECS is rate limiting enzyme of GSH biosynthesis (Xiang and Oliver, 1998). In As^V^ stressed plants, cysteine content and γ-ECS activity were enhanced that indicates the enhanced synthesis of GSH. Our results of GSH estimation also conferred the enhance level of GSH. NO treatment also enhanced the cysteine and γ-ECS. NO mediated enhancement of γ-ECS has been reported in *Medicago* by Innocenti et al. (2007). CS, involved at final step of cysteine synthesis, was directly correlated with the level of cysteine (*R* = 0.94 in root and *R* = 0.96 in shoot). GSH protects the cell from free metal ions by forming non-toxic complexes and facilitates their sequestration and GST catalyzes these conjugations (Jozefczak et al., 2012). In As^V^ stressed plants activity of GST enhanced. Previously, enhanced activity of GST was reported in *Arabidopsis* after Cu and Cd treatment in order to stimulate free metal binding (Jozefczak et al., 2012).

Ascorbate and reduced glutathione are important antioxidant molecules in plant system and indicates the redox state of cell. They serve as redox buffering agents in protoplast and protect the plasma membrane from oxidation (Innocenti et al., 2007). GSH/GSSG ratio is also important for maintaining redox state of the cell (Dixit et al., 2015b). In the present study, under higher dose of As^V^ stressed plants GSH/GSSG ratio was declined, while NO supplementation to As^V^ stressed plants resumed this ratio. Glutathione reductase plays a crucial role in maintaining the ratio of GSH/GSSG by converting GSSG to GSH. Despite of decreased ratio of GSH/GSSG, GR activity was enhanced in As^V^ stressed plants. It suggests that metal induced stimulation of GR was not sufficient to cope up with massive GSH consuming effects of metal, such as direct metal GSH binding, GSH oxidation, and PCs synthesis (Jozefczak et al., 2012). NO treatment also decreased the GSH level in shoot while GSH/GSSG ratio remained unchanged, therefore, lowered GSH level probably not attributed to oxidative stress but it may be a consequence of GSH nitrosylation (de Pinto et al., 2002). Ascorbate content also reduced under As^V^ stress and in NO treated plants. Reduced level of Asc under As stress is previously reported by Kumar et al. (2013).

Nitrate reductase activity enhanced under As^V^ stress as previously reported by Singh et al. (2015), but in NO treated plants, irrespective of presence of As^V^, NR activity was greatly diminished. In As^V^ stressed plants despite of enhanced NR activity there was no corresponding increase observed in nitrite level. The possible explanation for this may be that NO is directly serving as antioxidant and higher NO synthesized due to enhanced activity of NR got consumed in neutralization of free radicals produced due to As^V^, while during exogenous supplementation of NO, plants have stopped endogenous synthesis of NO.

## Conclusion

Nitric oxide ameliorated the As toxicity in rice by modulating antioxidant system and thiol metabolism. NO supplementation also significantly reduced the As accumulation in root and shoot and modulated the gene expression level of As^III^ transporters (*OsLsi1 and OsLsi2*). As and NO affected the GSH metabolism both at biosynthesis, i.e., cysteine, and consumption, i.e., PC and altered GSSG/GSH balance.

## Author Contributions

RT, DC, PT, PS, VP, SM^1^, and OD designed experiments and reviewed manuscript. AS and GD performed experimental work and prepared figures. AS and SM^2^ prepared the manuscript. AK and SD helped in elemental analysis. AK did data analysis. All authors have read and approved the manuscript.

SM^1^, Shekhar Mallick; SM^2^, Seema Mishra.

## Conflict of Interest Statement

The authors declare that the research was conducted in the absence of any commercial or financial relationships that could be construed as a potential conflict of interest.
