# Supplementary material for: Nitric Oxide Alleviated Arsenic Toxicity by Modulation of Antioxidants and Thiol Metabolism in Rice (Oryza sativa L.)
Source: Front Plant Sci. 2016 Jan 12;6:1272. doi: 10.3389/fpls.2015.01272 (PMC4709823; doi:10.3389/fpls.2015.01272)
Supplement: Supplementary file 1 [file Table_1.DOCX]

**Supplementary Table S-1:**Primer sequences of qRT-PCR analysis.

| *OsLsi1*F | GACTTCTTCCCTCCTCACCT |
| --- | --- |
| *OsLsi1*R | GCCGACGGCGTAGATCATCA |
| *OsLsi2*F | ATGAGTGAGCTTGCGTCGG |
| *OsLsi2*R | CAGGATTGGGAGGTCGATGGA |
| *OsFRDL1*F | GGCATTCCTTTTGTCGCTGG |
| *OsFRDL1*R | AGATAGCAGCCACACCAACC |
| *OsYSL2*F | TGTCTTGAGCTGTCTGCTGG |
| *OsYSL2*R | ATCCCTGTATTTGCGTGGCA |
| *OsIRO2*F | AAGCTCTACTCCTCCCTCCG |
| *OsIRO2*R | CTTCTGCAGCTCGGGTATGT |
| *OsNRAMP5*F | AAGAGGACGCCGACAAGTG |
| *OsNRAMP5*R | TGTGCCGGTAATAGTGGAGC |
| Rice actin F | GGAAGTACAGTGTCTGGATTGGAG |
| Rice actin R | TCTTGGCTTAGCATTCTTGGGT |
