# Supplementary figures and images for: Nitric Oxide Alleviated Arsenic Toxicity by Modulation of Antioxidants and Thiol Metabolism in Rice (Oryza sativa L.)
Source: Front Plant Sci. 2016 Jan 12;6:1272. doi: 10.3389/fpls.2015.01272 (PMC4709823; doi:10.3389/fpls.2015.01272)

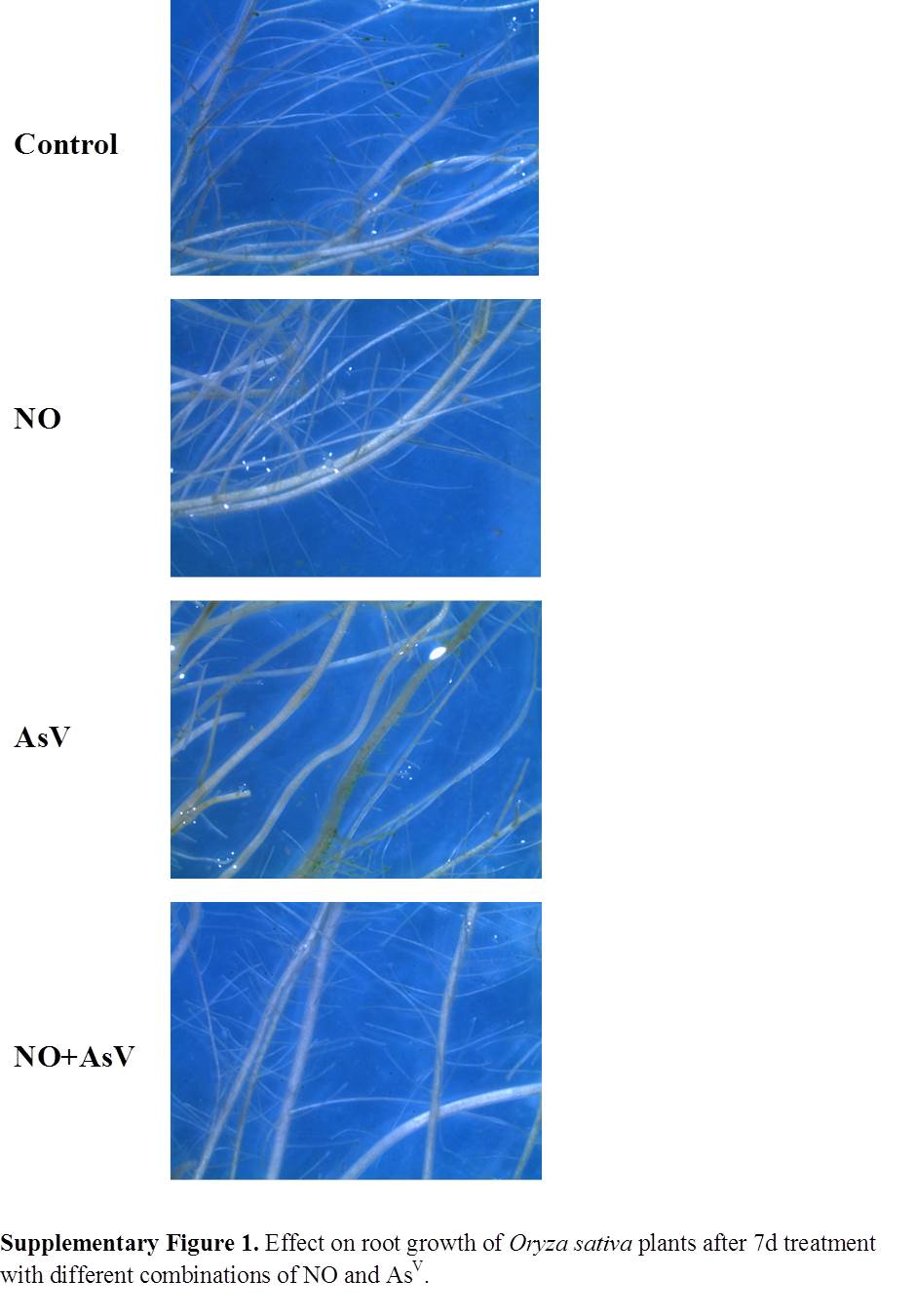

Supplement: Supplementary file 2 [file Image_1.JPEG]

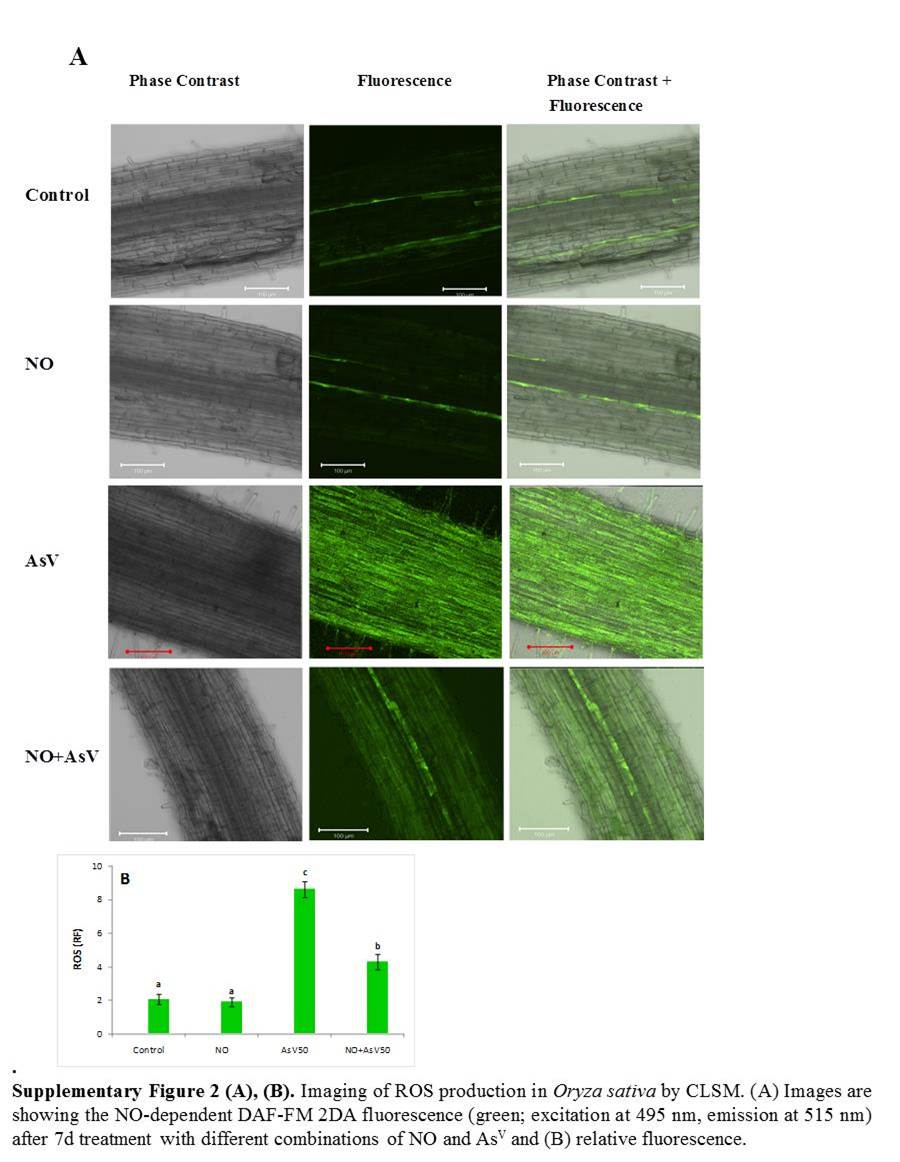

Supplement: Supplementary file 3 [file Image_2.JPEG]
